# Serum sickness-like reaction associated with cefazolin

**DOI:** 10.1186/1472-6904-6-3

**Published:** 2006-02-23

**Authors:** Michael Brucculeri, Marian Charlton, David Serur

**Affiliations:** 1 Division of Nephrology and Hypertension, New York Presbyterian Hospital – Weill Medical College of Cornell University, 525 East 68th Street, New York, NY 10021, USA; 2 The Rogosin Institute, New York Presbyterian Hospital – Weill Medical College of Cornell University, 525 East 68th Street, New York, NY 10021, USA

## Abstract

**Background:**

Although rare, serum sickness-like reactions have been documented to occur following the administration of many antibiotics. Cefazolin, a first generation cephalosporin, is a commonly prescribed antibiotic which is considered to be generally safe and well tolerated. There have been no prior reports linking this drug with sickness-like reactions. We report a probable case of serum sickness-like reaction following a single dose of cefazolin.

**Case Presentation:**

A 23 year old man with no significant past medical history was admitted to undergo a laparoscopic donor nephrectomy as part of a living-related renal transplant. One gram of intravenous cefazolin was administered perioperatively. The surgery was completed without complication and the remainder of his hospital course was uneventful. Ten days following discharge the patient developed fevers, painful and swollen joints, and a cutaneous eruption overlying his trunk and extremities. There was no evidence of systemic vasculitis. These clinical findings were most consistent with a serum sickness-like reaction. A brief course of corticosteroids and antihistaminergic therapy was initiated, and complete resolution of the patient's symptoms followed. The Naranjo probability scale indicated that this adverse drug event was probable.

**Conclusion:**

Serum sickness-like reaction may be associated with cefazolin therapy.

## Background

Serum sickness-like reactions (SSLR), although rare in clinical practice, have been documented to occur following the administration of many medications. While the pathophysiology which produces SSLR are not clear, they are constituted by an array of clinical findings including fever, cutaneous eruptions and arthralgias/arthritis. Literature reports have implicated several antibiotics including ciprofloxacin [1,2], minocycline[3], cefprozil[4], and cefaclor [5-7]. Herein, we report a probable case of SSLR following a single dose of cefazolin.

## Case Presentation

The patient, a 23 year old male with no significant past medical history, underwent left laparoscopic donor nephrectomy as part of a living-related renal transplant. On the day of surgery he was administered one gram of intravenous cefazolin which served as routine perioperative prophylaxis. Anesthesia was achieved with inhaled desflurane, along with fentanyl, propofol, and midazolam. Cisatracurium was employed for neuromuscular blockade. Post operatively, he received morphine sulfate, ketorolac, and ondansetron. His recovery was uneventful, and he was discharged home three days after his surgery with prescriptions for docusate sodium, acetaminophen, and hydrocodone. Upon discharge his serum creatinine was 1.8 mg/dL, WBC 10.0 per 10^9^/L, platelet count 197 per 10^9^/L, hemoglobin 13.4 g/dL. Eosinophils constituted 0.2% of WBC population.

Post operative day ten was marked by the emergence of fevers as high as 38.4 C, generalized weakness, and arthralgias involving bilateral knees, wrists, ankles, and metacarpophalangeal (MCP) joints. Simultaneously, a cutaneous eruption appeared which was initially limited to bilateral upper extremities but soon involved his trunk, back, face and lower extremities. Furthermore, additional MCP joints become swollen and tender in sequential order from medial to lateral. Upon questioning, he denied the presence of any gastrointestinal disturbance such as nausea, emesis, or diarrhea. On physical examination he was found to be afebrile. He appeared uncomfortable but was in no apparent distress. Oropharyngeal, pulmonary, and cardiac examinations were unremarkable. Although there was mild peri-incisional tenderness, his abdomen was soft and his surgical wounds appeared to be healing well with no evidence of local infection. There was no lymphadenopathy. Diffuse urticaria involving all extremities, trunk, back, neck, and face were noted. Nearly all his MCP joints were warm, swollen, and tender to touch. Of note, there appeared to be a sequential pattern of early MCP joint resolution as the medial joints that had been initially affected most, now appeared to be improving somewhat while the lateral ones were now most affected. In addition, the knees, ankles, wrists, and left metatarsophalangeal joint were also affected, although no effusions were appreciated upon physical examination. Repeat laboratory analysis was performed which revealed serum creatinine was 1.6 mg/dL, WBC 9.7 per 10^9^/L, platelet count 353 per 10^9^/L, hemoglobin 13.8 g/dL. Eosinophils constituted 2.6% of WBC population. Serum complement levels, immunoglobulin concentrations, and erythrocyte sedimentations rate were not obtained at this time. Urinalysis revealed trace albuminuria, with no hematuria or glycosuria. Microscopic examination of the urine revealed rare hyaline casts and the distinct absence of red or white cells and cellular casts.

Based on the aforementioned clinical findings, the absence of systemic vasculitis, and a history of a cephalosporin antibiotic exposure approximately two weeks earlier, a diagnosis of a SSLR was made. For five days, he was treated with prednisone 20 mg twice daily, loratadine 10 mg daily as well as famotidine 20 mg twice daily for H_2 _receptor blockade. Within 48 hours he noted near resolution of the urticaria as well as a significant improvement in the inflamed joints. He was reevaluated two weeks after this treatment and was found to have no further evidence of fevers, rash, or joint involvement. Antinuclear antibodies by enzyme immunoassay were not detected, serum complement levels were within the normal ranges for our laboratory as was serum IgE levels which were measured at 36.8 IU/mL.

## Discussion

Serum sickness-like reactions have been noted to occur in response to a variety of drugs, a majority of them being antibiotics[5]. Manifestations often include fever, rash, and joint inflammation, but generally lack generalized lymphadenopathy and evidence of systemic vasculitis such as glomerulonephritis [8]. Accordingly, the levels of circulating immune complexes and serum complement are often unaffected[5].

While the underlying pathophysiology remains unclear, SSLR continues to be documented as an adverse event for a small but growing number of drugs. To date there have been no published reports of serum sickness or SSLR following cefazolin administration. Although our patient did receive various other medications, only cefazolin belongs to a drug class which has repeatedly been implicated as a cause for SSLR. In the case just described, the Naranjo probability scale for adverse drug reactions indicates that cefazolin was the probable cause of SSLR[9].

## Conclusion

Several drugs have been connected to the development of SSLR including cephalosporin antibiotics such as cefaclor and cefprozil. We now report a probable case of SSLR in connection with cefazolin. Accordingly, we urge clinicians need to be aware of SSLR as a potential complication to this common therapy.

## List of abbreviations

C – Celsius

dL – deciliter

g – gram

IU – international units

L – liter

MCP – metacarpophalangeal

SSLR – serum sickness-like reaction

WBC – white blood count

## Competing interests

The author(s) declare that they have no competing interests.

## Authors' contributions

MB: Direct patient care, literature search, case review and summary, drafting of the original article. MC: Direct patient care, article conception and review. DS: Direct patient care, article conception, critical and extensive revision of article for important intellectual content, review and drafting of the original article. All authors read and approved the final manuscript and contributed equally to the manuscript.

## Pre-publication history

The pre-publication history for this paper can be accessed here:


